# Investigation of the Effect of Neck Muscle Active Force on Whiplash Injury of the Cervical Spine

**DOI:** 10.1155/2018/4542750

**Published:** 2018-04-04

**Authors:** Yu Yan, Jing Huang, Fan Li, Lin Hu

**Affiliations:** ^1^Research Centre of Vehicle and Traffic Safety, State Key Laboratory of Advanced Design and Manufacturing for Vehicle Body, Hunan University, Changsha, China; ^2^College of Automotive and Mechanical Engineering, Changsha University of Science & Technology, Changsha, China

## Abstract

The objective of the present study is to investigate the influence of neck muscle activation on whiplash neck injury of the occupants of a passenger vehicle under different severities of frontal and rear-end impact collisions. The finite element (FE) model has been used as a versatile tool to simulate and understand the whiplash injury mechanism for occupant injury prevention. However, whiplash injuries and injury mechanisms have rarely been investigated in connection with neck active muscle forces, which restricts the complete reappearance and understanding of the injury mechanism. In this manuscript, a mixed FE human model in a sitting posture with an active head-neck was developed. The response of the cervical spine under frontal and rear-end collision conditions was then studied using the FE model with and without neck muscle activation. The effect of the neck muscle activation on the whiplash injury was studied based on the results of the FE simulations. The results indicated that the neck active force influenced the head-neck dynamic response and whiplash injury during a collision, especially in a low-speed collision.

## 1. Introduction

Whiplash injuries occurring in car accidents are an increasing problem all over the world [1]; approximately 28–53% of traffic collision victims suffer this type of injury [2]. It continues to be a major health problem because of the long-term consequences [3]. Vehicle collision may cause sprains or strains to soft tissues in the neck and result in a variety of clinical manifestations, including headaches, dizziness, forgetfulness, and nerve root traits. These symptoms are collectively referred to as whiplash-associated disorders (WAD) [4]. However, the mechanism and location of whiplash injuries are still under investigation.

In the past few decades, many researchers conducted studies to investigate the mechanism of whiplash injuries via human volunteer tests [5, 6], mathematical models [7, 8], crash dummies [9], whole cadavers [10], and hybrid cadaveric models [11]. Luan [10] studied the kinematic responses and load patterns of human necks in a low-speed rear-end collision using cadaver tests. Krakenes et al. [12] evaluated the condition of the alar ligament in whiplash injuries using magnetic resonance imaging (MRI), which indicated that the alar ligament was vulnerable to whiplash injury and that MRI was a useful tool to assess the severity of neck injury. Fice and Cronin [2] investigated the occupant kinematic response and possible whiplash injuries of the upper cervical spine during a vehicle collision using a validated human head-neck finite element model. Based on Folksam's traffic injury survey, Jonsson et al. [13] studied the whiplash injury outcome of a front-seat occupant during a rear-end collision using a double paired comparison technique. Ivancic and Xiao [11] evaluated the biofidelity of a human FE model via comparisons with in vivo data and investigated the neck load and motion responses during simulated rear-end collisions, followed by studies of the mechanisms of whiplash injury and prevention methods. These studies revealed that the potential anatomical injury sites of the neck, facet joints, spinal ligaments, intervertebral discs, dorsal root ganglia (DRG), neck muscles, and vertebral arteries were vulnerable spots [1]. Brault et al. [14] studied the kinematic responses and injuries of neck muscles in a low-speed rear-end collision using volunteer experiments and found that neck muscles contracted rapidly during the collision and the prolonged muscle contraction would lead to potential muscle injury. Kumar et al. [15] carried out volunteer experiments to study the response of neck muscles when the head rotated during a side impact, indicating that the muscle force and the risk of muscle injury were reduced when the head was turned to the right or left in a side impact. The active force caused by the neck muscle contraction in a vehicle collision, especially in a low-speed collision, would have an important effect on the dynamic response of the human head and neck [16]. However, whiplash injuries as well as its injury mechanisms are rarely investigated in connection with neck restraint and active muscle forces, which would restrict the complete reappearance and understanding of the injury mechanism, There is still a need for better understanding of the whiplash injury mechanism for occupant injury prevention.

To better understand whiplash injury and its prevention mechanisms, the present manuscript described a numerical study with the objective of determining the effect of neck muscle activation on the head-neck dynamic response and whiplash injuries of the occupants in passenger vehicle front and rear-end collisions.

## 2. Methods and Materials

An active human head-neck FE model was developed and the mechanical property of the neck muscle was described via a three-element Hill-type model with both passive and active properties. The active head-neck model was then connected to the torso of a Hybrid III dummy to establish a mixed human model. The response of the cervical spine under frontal (8 g, 15 g, and 22 g) and rear-end (4 g, 7 g, and 10 g) collision conditions was studied using FE models with and without neck muscle activation. The effect of the neck muscle activation on the whiplash injury was then investigated based on the simulation results, the force-distraction response of the upper cervical ligament, the peak angle of the head, and the relative rotation angle of the cervical vertebrae, and whiplash injury criteria NIC, Nkm, and Nij were used as the analysis parameters.

### 2.1. Collision FE Model

#### 2.1.1. Active Head-Neck FE Model

**Figure 1:**
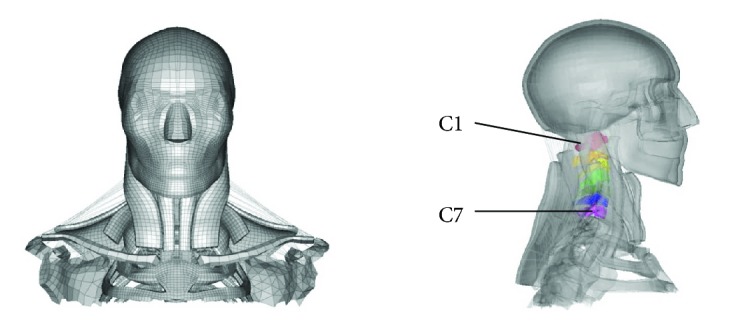
Active head-neck FE model.

An active head-neck FE model was developed as shown in Figure 1. The basic model was developed at Hunan University based on the human anatomy structure of a 50th percentile adult male, which was validated against experiment data in frontal, rear-end, and side impact conditions [17, 18], and the basic model was subsequently improved and validated by Zhang and Yang [19], Li et al. [20], and Huang et al. [21]. The updated active head-neck FE model represented all essential anatomical features of a 50th percentile male head and neck, including the scalp, skull with outer table, diploe, inner table, dura mater, falx cerebri, tentorium, falx cerebelli, pia, cerebral spinal fluid (CSF), cerebrum, cerebellum, brain stem, ventricles, cervical vertebrae, and three-dimensional neck muscles with passive and active properties.

The whole model consists of 554,154 elements; 620,899 nodes; and 427 parts, including 442,094 solid elements; 107,620 shell elements; 59 spring elements; and 4381 beam elements. The neck was modelled with 52 components, including muscles, deformable vertebrae, cartilage, ligament, and intervertebral discs. The attachments of the neck muscles were distributed on the sternum, ribs, and thoracic vertebrae according to the anatomic structures.

**Figure 2:**
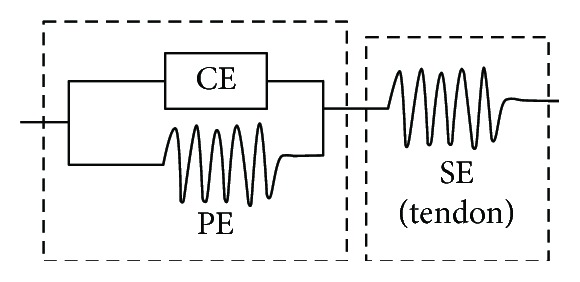
Schematics of the three-element Hill-type muscle model.

The mechanical property of the neck muscle was described via a three-element Hill-type model with both passive and active properties, as shown in Figure 2.

The model comprises a contractile element (CE) and two nonlinear spring elements: one in series (SE) and one in parallel (PE). The PE element represents the stiffness of the passive muscle tissue and is modelled using nonlinear characteristics. The SE element represents the tendons by which the muscle is connected to the skeletal structure. The CE element generates the active force when the muscle is activated. The total force generated by a muscle is the sum of the forces generated by all components.

**Figure 3:**
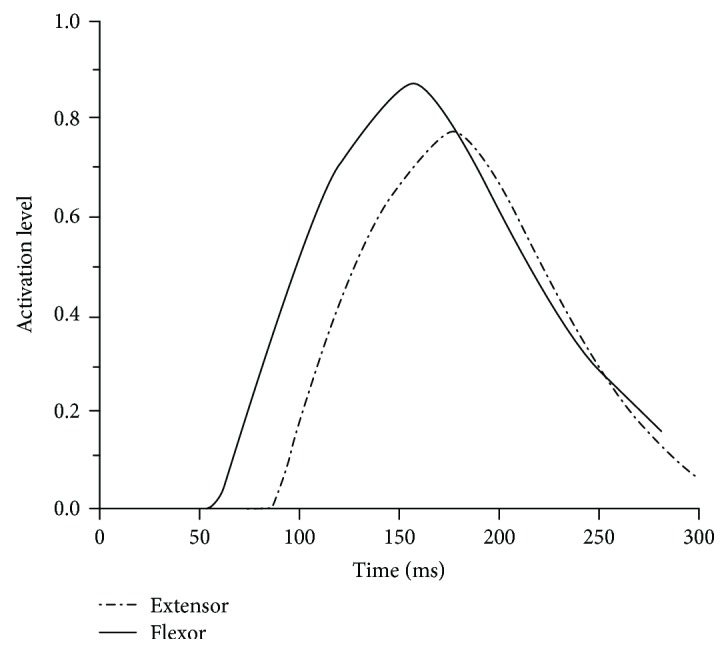
The curves for setting the activation level and trigger time for the neck muscles.

The solid elements were used to simulate the passive response property of the neck muscles, and the material property was described using the Ogden rubber model (MAT_OGDEN_RUBBER) [22]. The active response property was simulated by the beam elements, and the material property was defined with the material model in DYNA (MAT_MUSCLE) [22]. Muscle activation was triggered by the motion of the lower cervical spine, as suggested by Szabo and Welcher [23], and the main extensor muscles would be activated prior to the flexors; the activation levels were not the same, and a higher activation level corresponded to the larger force produced by the muscles. In this manuscript, the trigger time and activation level of the extensor and flexor were set as suggested by Kumar et al. [24], as shown in Figure 3, and the passive properties of the musculature were defined to be the same in two groups of simulations.

#### 2.1.2. The Mixed Human FE Model in the Seated Posture

The mixed human FE model was composed of the active head-neck model mentioned above as well as the torso of Hybrid III dummy model, and its biofidelity was validated with the data from volunteer experiments [20, 25]. The head and neck of the Hybrid III dummy were removed, the first thoracic vertebra (T1) of the active head-neck model was overlapped and placed on the T1 of the Hybrid III dummy model, and the two T1 were rigidly connected by the keyword CONSTRAINED_RIGID_BODIES.

#### 2.1.3. The Collision FE Model

**Figure 4:**
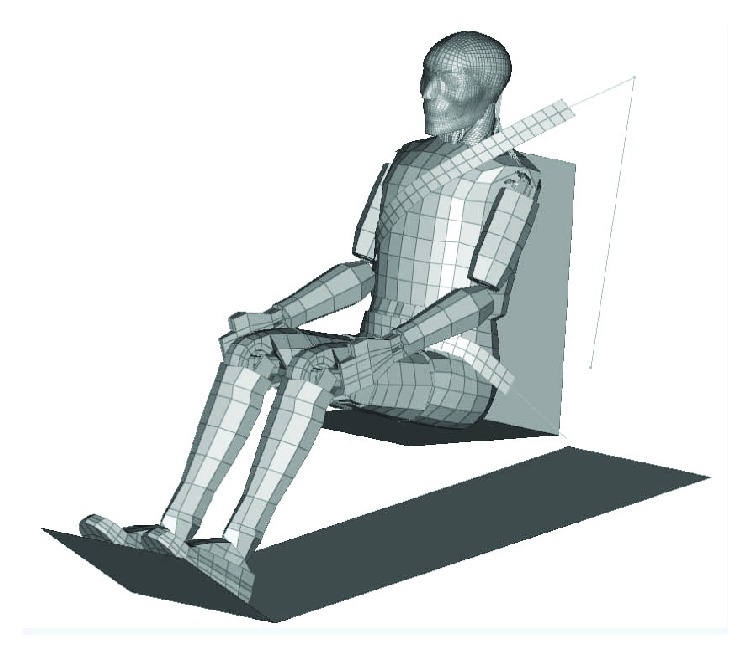
Collision FE model.

The collision FE model was shown in Figure 4; it included the mixed human FE model in a sitting posture, a simplified car as well as its seat, and an occupant restraint system. The simplified car model was modelled using two planar elements, which represent the car floor and the foot pedal and were defined as rigid materials, with the pedal 45° from the horizontal. According to the test configuration of the human volunteer collision test carried out at the Japan Automobile Research Institute (JARI) [26], the seat cushion angle was 10° from the horizontal, and the seatback angle was 20° from the vertical. The human model was constrained by an occupant restraint system, which was composed of an anchor point, slip ring, retractor, and webbing belt. The webbing belt was simulated by a combination of a 2D element and 1D element. The 2D element belt contacted with the human body to present the contact and relative slide between the seat belt and dummy. The 1D element belt was defined with the keyword ELEMENT_SEATBELT, which can simulate sliding along the slip ring and the characteristics of the retractor. The biofidelity of the collision FE model was validated against the volunteer experiments [25, 27].

### 2.2. Virtual Experimental Methodology

**Figure 5:**
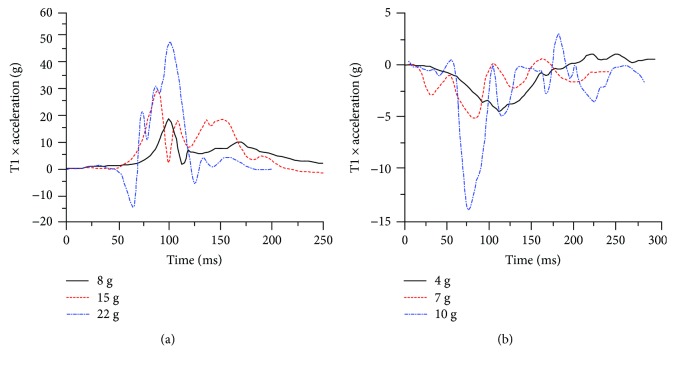
T1 acceleration in the anterior-posterior direction for (a) frontal impacts and (b) rear-end impacts.

Although it has been traditionally reported that rear impacts account for most cases of whiplash injury [28, 29], a large epidemiologic study has suggested that rear and frontal collisions account for whiplash injury in roughly equal proportions [15]. Therefore, frontal and rear-end collisions were both involved in the present study, and according to a previous study [2], the response of the cervical spine was investigated at increasing impact severities in both frontal (8 g, 15 g, and 22 g) and rear (4 g, 7 g, and 10 g) impact conditions using the FE model with and without neck muscle activation. Previous studies [2, 3, 30] indicated that the kinematic response of the upper cervical spine in vehicle collisions was related to possible whiplash injury, and the acceleration of T1 in the anterior-posterior direction obtained from the corresponding cadaver experiments was used as the initial condition and input boundary of the virtual experiments [2], as shown in Figure 5.

### 2.3. Data Analysis

The excessive loads, displacements, and head-T1 relative acceleration and velocity would cause neck injuries [31]. The neck injury criterion (NIC) is based upon the head-T1 relative acceleration and velocity; the neck protection criterion (Nkm) and the normalized neck injury criterion (Nij) are functions of the dynamic loads at the occipital condyles [32], and they can be used as whiplash injury criteria to predict neck injuries and to evaluate the effectiveness of safety systems in reducing the risk of injury. Therefore, in the present study, the neck injury indexes NIC, Nkm, and Nij, as well as the force-distraction response of the upper cervical ligament, the peak rotation angle of the head, and the relative rotation angle of the cervical vertebrae under each collision severity, were calculated and studied.

## 3. Results

The effect of neck muscle activation on neck whiplash injury was investigated based on the results from the FE simulations under different collision severities.

### 3.1. Ligament Distractions of the Upper Cervical Spine

**Figure 6:**
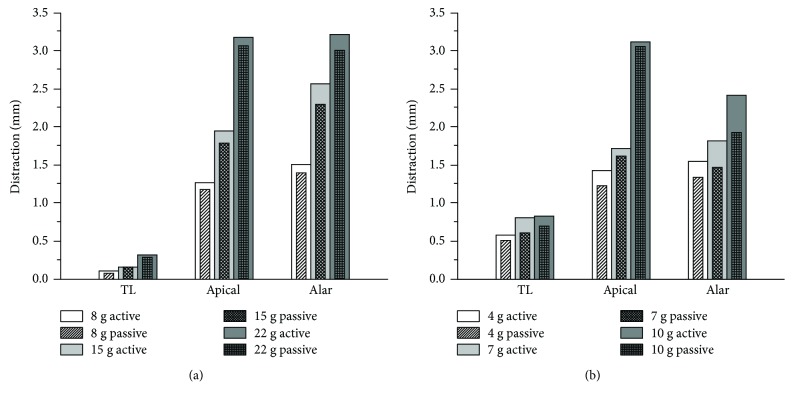
Disruption of ligaments under different collision severities: (a) frontal impacts and (b) rear-end impacts.

In the literature [2], the upper cervical spine ligaments, especially the alar ligament, have been identified as a potential whiplash injury location. Therefore, in the present study, the disruptions of the transverse ligament (TL), the alar ligament (alar), and the apical ligament (apical) of the upper cervical spine under different collision severities were calculated, as shown in Figure 6.

In the frontal and rear-end collisions under all collision severities, the ligament disruptions of TL, alar, and apical in the active model showed a larger value compared with those in the passive model, and the disruption increased with increasing collision severity. In frontal collisions, the alar ligament suffered the largest disruption of 3.22 mm when the collision acceleration was up to 22 g, and the largest disruption of the apical ligament and transverse ligament was 3.18 mm and 0.32 mm, respectively. In rear-end collisions, the apical ligament suffered the largest disruption of 3.12 mm when the collision acceleration was up to 10 g, and the largest disruption of the alar ligament and transverse ligament was 2.42 mm and 0.83 mm, respectively.

### 3.2. Peak Head Rotation and Relative Vertebral Rotations

The peak head rotations and relative vertebral rotations for each collision severity are shown in Table 1.

The peak head rotation and the relative vertebral rotations increased with increasing collision severity during the frontal and rear-end collisions. The rotations of the active model showed the same or a smaller value compared with those of the passive model under the same collision severity, except the C3-C4 angle of the frontal collision under the collision acceleration of 22 g and the C1-C2 angle of the rear-end collision under the collision acceleration of 10 g.

### 3.3. Whiplash Injury Criteria

The maximum values of NIC, Nkm, and Nij of the active model and passive model under different collision severities were compared in Table 2. All the whiplash injury criteria increased with increasing collision acceleration in frontal and rear-end collisions, and the active muscle force increased the NIC peaks and Nkm peaks compared with the passive model. The changing law of Nij was different from NIC and Nkm; the attendance of active muscle force increased the Nij peaks in frontal collisions but decreased the Nij peaks in rear-end collisions.

**Figure 7:**
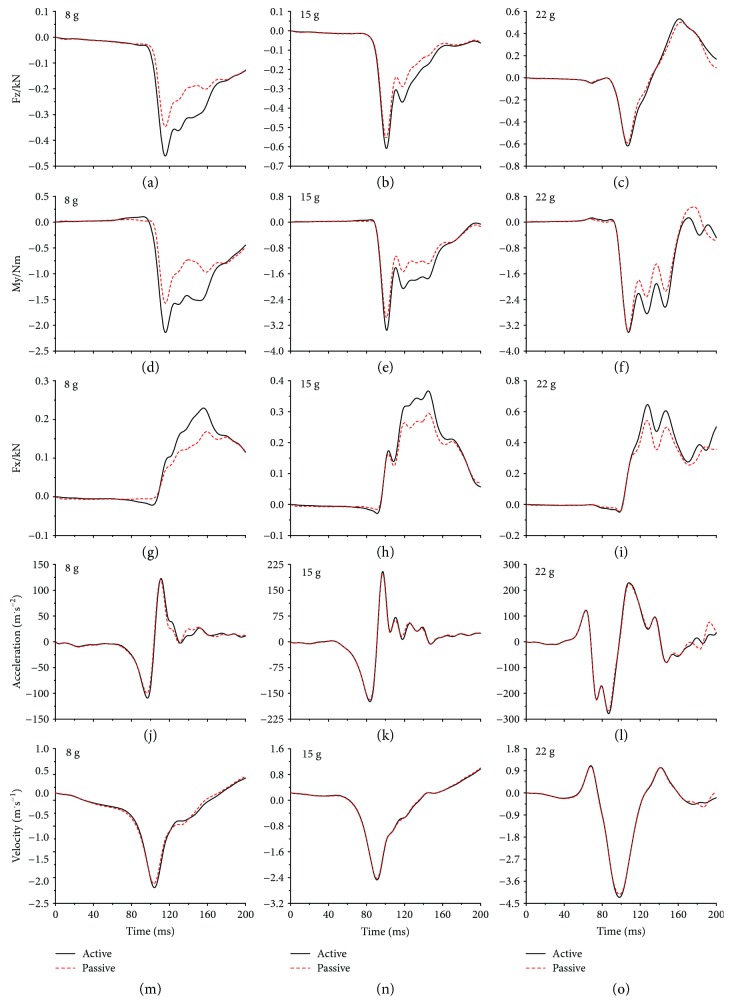
The calculation parameters of neck injury criterion in frontal collisions.

**Figure 8:**
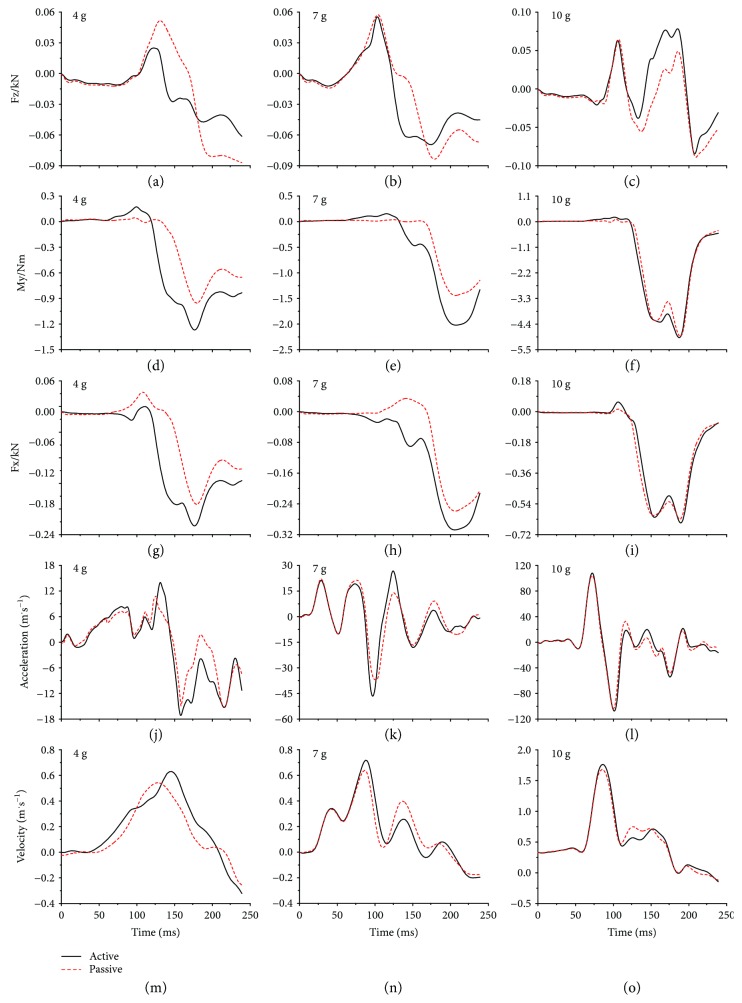
The calculation parameters of neck injury criterion in rear-end collisions.

For the injury criteria, NIC, Nkm, and Nij are based upon neck shear force (Fx), axial force (Fz), and moment (My), as well as the relative horizontal acceleration and velocity between the head and T1 CoMs; the time history curves of these parameters of the active model and passive model under different collision severities were compared in Figures 7 and 8.

## 4. Discussion

The whiplash-related responses of the upper cervical spine and injury criteria were computed and compared during simulated frontal and rear-end collisions with and without the active muscle force. Although the mechanisms causing the whiplash injuries are not fully known, it is possible to identify parameters influencing the whiplash injury risk.

From the ligament distractions of the upper cervical spine illustrated in Figure 1, we observed that the alar ligament and apical ligament were more sensitive to the collision severity, whose disruptions were much larger than those of the transverse ligament under the same collision severity both in frontal and rear-end conditions. For example, in a 15 g frontal collision, the disruption of the transverse ligament of the active model was only 0.16 mm, while that of the alar ligament was 2.57 mm, which is sixteen times as large as the disruption of the transverse ligament. This finding supports the clinical MRI findings [33] and coincides with the conclusion in the literature [2] that the alar ligament and apical ligament have been identified as a potential location of whiplash injury. On the another hand, the disruptions of all the three ligaments in a 4 g rear-end collision (TL 0.58 mm, alar ligament 1.55 mm, and apical ligament 1.43 mm) were larger than the disruptions in an 8 g frontal collision (TL 0.11 mm, alar ligament 1.51 mm, and apical ligament 1.27 mm), which indicated that the incidence of whiplash neck injury is highest in rear-end collisions compared to other collision configurations. The neck active muscle force increased the ligament disruptions under all collision severities both in frontal and rear-end collisions, especially the alar ligament, which is in agreement with the MRI findings that 72% of whiplash patients in frontal collisions and 58% of rear-end collisions exhibited potential alar damage [33].

The cervical spine (C1–C7) is the most manoeuvrable region of the spine. Table 1 listed the peak head rotation and relative vertebral rotations, and the head rotation and relative intervertebral rotations were found to increase with increasing collision severity for both frontal and rear-end collisions. In most cases, the active muscle force reduced the rotation angles of the head and angles between the cervical vertebrae. There were two special cases; they were the C3-C4 angle of frontal collision under the 22 g collision acceleration and the C1-C2 angle of rear-end collision under the 10 g collision acceleration. It should be noted that the influence of active muscle force on relative vertebral rotations in frontal collisions decreased with the collision severity. When the collision acceleration was up to 22 g, the difference between the relative vertebral rotations of the active model and the passive model was very small. It can be assumed that in a high-severity collision, the influence of active muscle force on the relative vertebral rotations can be neglected, so the special case is acceptable where C3-C4 angle of the active model was 0.04° larger than that of the passive model. It is suggested that when investigating the intervertebral neck injury, the neck active muscle force must be involved. From Figure 1, it can be observed that the attendance of active muscle force made the distraction of the alar ligament increased 26% under the 10 g rear-end collision, and according to the anatomical structure, the alar ligament damage would affect the stability of the atlantoaxial joint (C1-C2). This may explain the reversed situation of C1-C2 angle of rear-end collision under the 10 g collision acceleration.

The injury criteria NIC, Nkm, and Nij are based upon neck loads and head-T1 relative acceleration and velocity; they are used as the whiplash injury criteria to predict neck injuries in the present study. Table 2 showed the whiplash injury criteria for each collision severity; the results indicated that a higher collision severity caused a higher risk of injury without incident. The Nij of rear-end collisions under three collision severities was very small (the order of magnitude is 10^−2^) and showed small changes (the order of magnitude is 10^−3^) with collision severity and attendance of active muscle force. This finding supports the rule that the Nij is suited to predict neck injuries in frontal collisions. On the other hand, the NIC of frontal collisions under three collision severities was very large, even under the 8 g collision acceleration, where the Nkm and Nij were far lower than the tolerance threshold of 1, the NIC already exceeded the tolerance threshold of 15 m^2^/s^2^. In addition, the NIC was computed using the relative horizontal acceleration and velocity between the head and T1 CoMs, whose time history curves were shown in Figures 7(j)–7(o) and Figures 8(j)–8(o); we observed that the attendance of active muscle force increased the relative horizontal acceleration and velocity peaks in rear-end collisions, especially in lower-severity rear-end collisions, while nearly did not cause any change in frontal collisions. This finding supports the rule that NIC is suited to predict the neck injuries in a rear collision.

Figure 6 and Table 1 revealed that the active force generated by the neck muscles increased the ligament disruption and decreased the relative vertebral rotations. Meanwhile, active force applied an additional effect on the cervical spine to affect the relative horizontal acceleration and velocity between the head and T1 CoMs, shear force (Fx), axial force (Fz), and moment (My), as shown in Figures 7 and 8. This effect increased the whiplash injury criteria Nkm peaks in both frontal and rear-end collisions, NIC peaks in rear-end collisions, and Nij peaks in frontal collisions; the same tendency can be observed in their calculating parameters, as shown in Figures 7(a)–7(i) and Figures 8(d)–8(o). In rear-end collisions, the neck injuries mainly follow the tension-extension mechanism; due to the constraint of the headrest, the extension movement of the upper cervical spine was held back, and the force generated by the neck muscles offset certain tension suffered by the cervical spine, which then decreased the axial force (Fz), as shown in Figures 8(a)–8(c), so that the active model suffered a lower Nij compared to the passive model. Ivancic and Sha [34] suggested neck injuries may occur at a peak Nkm of 0.33 or Nij of 0.09, and whiplash injuries may occur even if head-T1 motions are small. It should be noted that with this suggested whiplash injury threshold, the attendance of active force would change the whiplash injury prediction results in some collisions. For example, with the Nij of a frontal 8 g collision and the Nkm of a rear-end 4 g collision, due to the attendance of the neck active muscle force, the risk of whiplash injury changed from no to yes. Of course, the limitations of our study should be considered: the trigger time and activation level of the muscles are assumed to be the same, while they are actually dependent on the collision severity and collision type. Although the simulation results may not reveal the real injury risk, the tendency is very clear that the force generated by the neck muscles will influence the head-neck dynamic response as well as the neck injury risk. Future studies that concentrate on the actual trigger time and activation level might indicate dimensions of fall protection that could fruitfully be developed by focused research on this mechanism of injury and injury prevention.

## 5. Conclusions

A detailed and validated mixed FE model of a 50th percentile male was used to investigate the effect of neck muscle active force on whiplash injury of the cervical spine. The whiplash-related response of the cervical spine and injury criteria were computed and compared under frontal (8 g, 15 g, and 22 g) and rear-end (4 g, 7 g, and 10 g) collisions. The different results between the active model and passive model were observed, and the active force generated by the neck muscles increased the ligament disruption, decreased the relative vertebral rotations, and increased the injury criteria peaks. This revealed that the neck active force would influence the head-neck dynamic response and whiplash injury risk during a collision. Compared with that of a high-speed collision, the effect of active muscle force is more significant in a low-speed collision. This suggests that when investigating the intervertebral neck injury and the neck injury risk in a low-speed collision, the effect of neck active muscle force must be involved.

## Figures and Tables

**Table 1:** Peak head rotations and relative vertebral rotations for each collision severity (flexion is positive rotation).

Rotations (deg.)	8 g		15 g		22 g	
Frontal collision severity	Passive	Active	Passive	Active	Passive	Active
Head	53.06	52.58	73.68	72.53	114.32	111.61
C1-C2	2.98	2.92	3.85	3.48	6.86	6.73
C2-C3	6.71	6.53	9.25	9.04	14.61	14.55
C3-C4	5.73	5.42	7.24	7.19	10.36	10.40
C4-C5	6.88	6.35	9.45	9.40	13.58	13.57
C5-C6	8.65	8.62	11.07	11.04	13.80	13.71
C6-C7	9.92	9.83	13.16	13.02	18.24	18.14

Rear-end collision severity	4 g		7 g		10 g	
Rotations (deg.)	Passive	Active	Passive	Active	Passive	Active
Head	−46.76	−43.37	−62.27	−59.48	−97.00	−95.94
C1-C2	−7.63	−7.15	−13.69	−13.17	−15.47	−16.90
C2-C3	−5.12	−5.12	−7.94	−7.20	−11.69	−11.44
C3-C4	−4.88	−4.04	−6.13	−5.52	−9.64	−9.43
C4-C5	−3.63	−3.12	−3.78	−3.59	−4.98	−4.57
C5-C6	−4.65	−4.33	−4.04	−3.54	−7.26	−6.99
C6-C7	−4.98	−4.73	−5.92	−5.45	−8.87	−8.22

**Table 2:** Whiplash injury criteria for each collision severity.

Frontal collision	8 g		15 g		22 g	
	Passive	Active	Passive	Active	Passive	Active
NIC	26.58	27.18	44.39	46.72	53.31	54.72
Nkm	0.21	0.29	0.36	0.46	0.67	0.8
Nij	0.08	0.11	0.13	0.15	0.14	0.15

Rear-end collision	4 g		7 g		10 g	
	Passive	Active	Passive	Active	Passive	Active
NIC	3.04	3.17	7.31	9.18	20.62	22.41
Nkm	0.32	0.39	0.43	0.58	0.78	0.8
Nij	0.023	0.018	0.022	0.019	0.024	0.023
